# Draft Genome Sequence of Enterococcus faecalis 1351, Isolated from a Mastitis-Affected Camel in Isiolo County, Kenya

**DOI:** 10.1128/MRA.00143-21

**Published:** 2021-04-15

**Authors:** Jesca Ochieng, Nimmo Gicheru, Abwao Stephen Indieka, Monicah Maichomo, O. Hezron Wesonga, Edwin Murungi

**Affiliations:** a Biochemistry and Molecular Biology Department, Egerton University, Njoro, Kenya; b Veterinary Science Research Institute, Kenya Agricultural and Livestock Research Organization, Nairobi, Kenya; c Department of Medical Biochemistry, Kisii University, Kisii, Kenya; University of Maryland School of Medicine

## Abstract

Enterococcus faecalis causes mastitis disease in livestock, leading to massive economic losses. Sequencing of isolates obtained from resource-poor regions will facilitate the design of novel sensitive diagnostics and efficacious vaccines. We announce the draft genome of E. faecalis strain 1351, which was obtained from a camel in Isiolo County, Kenya.

## ANNOUNCEMENT

The Gram-positive bacterium Enterococcus faecalis is one of the etiological agents of environmental mastitis, which afflicts livestock in sub-Saharan Africa, leading to substantial economic losses. In the arid and semiarid regions of northern Kenya, where pastoralists depend largely on camel meat and milk for their livelihoods, environmental mastitis poses a major economic challenge. Due to rampant misuse of common antibiotics in the management of mastitis, the emergence of E. faecalis as a multidrug-resistant pathogen has been reported (1, 2). Therefore, development of novel diagnostics and vaccines for E. faecalis infections is imperative. Achievement of this vital goal requires sequencing of strains isolated from different sources and geographical locations.

E. faecalis strain 1351 (k 10939) was isolated from a sample that was part of 696 milk samples obtained from 217 camels that had been confirmed to have mastitis using the California mastitis test (CMT). The samples were obtained from Isiolo County, Kenya (which lies between latitudes 0°05′S and 20°N and between longitudes 36°50′W and 39°50′E), cultured on blood agar enriched with 5% sheep blood, and then incubated at 37°C for 24 h. A single plate with small white colonies that showed no hemolysis and were catalase negative, indicative of E. faecalis, was selected. A single colony was streaked onto Edward’s agar and incubated at 37°C for 24 h. A 10-ml liquid culture was prepared using tryptone soy broth (Oxoid) at 37°C for 48 h, and the bacterial pellet was obtained by centrifugation at 3,000 × *g* for 15 min.

Sequencing was performed at the Technology Center for Genomics and Bioinformatics, University of California, Los Angeles (Los Angeles, CA, USA). The pellet was lysed using 100 μl phosphate-buffered saline (PBS) and 200 μl MagNA Pure lysis buffer (Roche Applied Science, USA). Genomic DNA was extracted using the MagNA Pure compact nucleic acid isolation kit I (Roche Applied Science) and was quantified using a Qubit fluorometer. DNA was fragmented using an Illumina fragmentation enzyme, and indices were attached by PCR. Library sequencing with the Illumina HiSeq 3000 platform produced 12,769,894 reads (250 bp). Adaptors and low-quality sequences were trimmed using Cutadapt v1.9.1 (3). Read quality was ascertained using FastQC v0.11.9 (4), and assembly was performed using SPAdes v3.13.2 (5) with the “only-assembler” option for k values of 55, 77, 99, and 127. Assembly quality was evaluated using QUAST v5.0.2 (6). The genome was initially annotated using PATRIC v3.6.3 (7) and was reannotated using Prokaryotic Genome Annotation Pipeline (PGAP) v4.11 (8). Unless otherwise mentioned, default parameters were used for all software tools.

The E. faecalis 1351 draft genome is 2,909,623 bp, assembled into 168 contigs, and has a GC content of 37.57%. The genome coverage was 12×, and the *N*_50_ value was 292,072 bp. The PGAP annotation identified 5,342 protein-coding genes, 68 tRNAs, and 14 rRNAs (2 complete and 12 partial rRNAs). The PATRIC annotation revealed 2,912 proteins, and *in silico* analysis revealed 5 novel vaccine candidates, including a cell wall surface anchor family protein, a putative lipoprotein, a 5′-nucleotidase, and 2 hypothetical proteins.

### Data availability.

This whole-genome shotgun project has been deposited in DDBJ/ENA/GenBank under the accession number JACTAL000000000. The version described in this paper is version JACTAL010000000. Raw sequence reads have been deposited under the accession number SRR13638389.
